# An Overview of Pre-Analytical Factors Impacting Metabolomics Analyses of Blood Samples

**DOI:** 10.3390/metabo14090474

**Published:** 2024-08-28

**Authors:** Amy Thachil, Li Wang, Rupasri Mandal, David Wishart, Tom Blydt-Hansen

**Affiliations:** 1 Department of Pediatrics, BC Children’s Hospital, University of British Columbia, Vancouver, BC V6T 1Z4, Canada; 2 Department of Pathology and Laboratory Medicine, Faculty of Medicine, University of British Columbia, Vancouver, BC V6T 1Z4, Canada; 3 BC Children’s Hospital Research Institute, Vancouver, BC V5Z 4H4, Canada; 4 Faculty of Science—Biological Sciences, The Metabolomics Innovation Centre, University of Alberta, Edmonton, AB T6G 2R3, Canada; 5 Department of Laboratory Medicine & Pathology, Faculty of Science—Biological Sciences, The Metabolomics Innovation Centre, University of Alberta, Edmonton, AB T6G 2R3, Canada; 6 Division of Nephrology, Department of Pediatrics, BC Children’s Hospital, University of British Columbia, Vancouver, BC V6T 1Z4, Canada

**Keywords:** metabolomics, blood, sample processing, pre-analytical, protocol

## Abstract

Discrepant sample processing remains a significant challenge within blood metabolomics research, introducing non-biological variation into the measured metabolome and biasing downstream results. Inconsistency during the pre-analytical phase can influence experimental processes, producing metabolome measurements that are non-representative of in vivo composition. To minimize variation, there is a need to create and adhere to standardized pre-analytical protocols for blood samples intended for use in metabolomics analyses. This will allow for reliable and reproducible findings within blood metabolomics research. In this review article, we provide an overview of the existing literature pertaining to pre-analytical factors that influence blood metabolite measurements. Pre-analytical factors including blood tube selection, pre- and post-processing time and temperature conditions, centrifugation conditions, freeze–thaw cycles, and long-term storage conditions are specifically discussed, with recommendations provided for best practices at each stage.

## 1. Introduction

Metabolomics is an emerging discipline in the field of “omics” that aims to analyze the collection of small molecules comprising the metabolome. Metabolites are produced downstream of energy processes and, when considered in relation to one another, produce a snapshot of metabolism at a particular point in time. The metabolite milieu carries with it information from genetic, epigenetic, and environmental influences [1,2] and is readily quantified in biofluids [2]. Blood and urine are frequently used in metabolomics research, but interest exists in analyzing additional fluids, including saliva and sweat [1,3]. The integrated physiological signal captured by the metabolome provides insight into the biological systems driving states of health and disease [2], making metabolomics a valuable platform for biomarker discovery. Previous work has identified candidate metabolic markers of cancer cell metabolism [4,5], kidney disease progression [6,7], neurological disorders [8], and stroke [9,10]. Other studies have shown the utility of metabolomics as a tool for monitoring responses to pharmacotherapies [11]. Our research group previously conducted metabolomics analyses to study acute kidney injury within the pediatric population [12] and evaluated metabolomics as a platform for detection of rejection outcomes following kidney transplantation [13,14]. By characterizing the metabolic signatures underlying these complex phenotypes, metabolomics can allow for the development of targeted therapies and personalized treatments [15].

Sample mishandling, lack of quality control during testing, flaws in experimental design, and random errors can influence measured metabolite profiles and have serious implications, especially for biomarker discovery. The range of analytes quantified in metabolomics research increases the likelihood of error and bias in downstream analysis work. The dynamic nature of the metabolome makes it particularly vulnerable to modification during the pre-analytical phase. The pre-analytical phase encompasses the collection, processing, and storage of the biosample of interest prior to being analyzed by liquid chromatography–mass spectrometry (LC-MS), gas chromatography–mass spectrometry (GC-MS), or nuclear magnetic resonance (NMR). Pre-analytical handling can introduce variation that is not representative of the original metabolic state. This is most evident in blood samples (serum and plasma), which contain a metabolically active cellular component prior to centrifugation. Variation introduced by sample handling can make it challenging to properly compare findings reported by different research groups.

Implementing Standard Operating Procedures (SOPs) at each step of sample processing is imperative to advancing the field of metabolomics. SOPs allow for large-scale, multi-center metabolomics analyses and robust intercomparison of research findings. SOPs are required to dictate optimal handling conditions and to standardize the preparation of samples intended for biobanking and subsequent utilization in metabolomics research. This review is intended to provide an up-to-date summary of the available literature on pre-analytical considerations to optimize the quality of metabolomics research using blood samples. For each stage of the pre-analytical phase, we provide recommendations for best practices. Research groups are encouraged to reference these guidelines when developing standardized workflows for blood metabolomics analyses. These guidelines may also be referenced when developing SOPs for biobanking to ensure optimal sample quality in downstream analytical work.

## 2. Choice of Matrix

### 2.1. Serum vs. Plasma

Serum and plasma are both acceptable matrices for blood metabolomics research. Although both originate from whole blood, differences in processing requirements can alter the metabolome composition detected in each biofluid. To generate serum from whole blood, serum collection tubes with or without clot activators need to be exposed to room temperature (RT) for 30–60 min to allow the cellular fraction to clot, followed by centrifugation to separate the liquid phase from cell and protein sediment [16,17]. Samples held for shorter durations prior to centrifugation are expected to retain cellular elements, whereas samples held for longer durations contain artefacts of cell lysis [16,18]. In contrast, plasma collection tubes contain anticoagulation agents and must be immediately centrifuged or maintained at a cool temperature until centrifugation [17,19,20,21]. As it relates to metabolomics analyses, it is important to consider whether serum and plasma are equivalent representations of the blood metabolome archetype.

Although there is no consensus as to which is the superior biofluid for metabolomics research, the existing literature illustrates measurable differences between the plasma and serum metabolomes. Several metabolite classes, including free fatty acids [22], acyl-carnitines [22], lysophosphatidylcholines [22,23], sphingomyelin [22], peptides [24,25], glucose [23,26], lactate [26], pyruvate [27,28,29], carbohydrates [27], amino acids [23,27], and many other organic molecules [27], have been shown to differ in concentration between serum and plasma (Table A1). The serum metabolite measurements that are most elevated relative to plasma include diacylphosphotidylcholines [23], lyso-phosphatidylcholines (C16:0, C17:0, C18:0, and C18:1) [23], serine [23,30], phenylalanine [23,28], glycine [23,28], charged peptide analytes [24,25], lactate [28], arginine [31], taurine [31], and serotonin [31]. Fewer metabolites are reported as having generally higher concentrations in plasma, but these include lipoproteins [26], tyrosine [30], urea [30], aspartic acid [30], and pyruvate [27,28]. Yu et al. [23] conducted a particularly well-designed analysis, measuring the concentration of 163 metabolites in fasting plasma and serum samples from a large number of individuals (*n* = 377). Pre-analytical conditions were kept consistent, with differences being permitted only for their differential preparation. Using a flow injection analysis–mass spectrometry (FIA-MS) approach, 104 metabolite concentrations were found to be higher in serum, with 9 concentration differences being greater than 20%. Serum was found to be more sensitive to group differences, which led to a greater number of metabolite concentration differences between patients with type 2 diabetes and healthy individuals. A similar trend was observed when distinguishing between sexes and between smokers and non-smokers. In their analysis, plasma was found to offer greater reproducibility as a matrix. Paired plasma samples had a mean correlation coefficient of 0.83 compared to 0.80 in serum (*p* = 0.01).

It is important to note that the existing body of literature is varied, making it difficult to ascertain definitive trends for individual metabolite concentrations. This is evident in the reporting of amino acid profiles in serum and plasma. Multiple reports suggest that amino acids differ in concentration between serum and plasma [23,27,28,29,30,31]. However, Liu et al. [22] found that most amino acids showed no significant concentration differences between paired serum and plasma obtained from 10 healthy volunteers. Their analysis may have been underpowered to show small differences, but these results speak to the inconsistencies observed in the existing literature. Moreover, no consensus exists as to which amino acids are most different between the two matrices. This exemplifies the fact that whilst serum and plasma profiles are discernibly different, we cannot draw conclusions related to specific metabolites or metabolite classes.

The generally higher concentration of certain metabolites in serum samples likely derives from the prolonged exposure to RT during pre-processing and the lengthy contact time of the cellular fraction with the fluid phase [16]. Indeed, the distinction between serum and plasma samples is defined by the former having gone through the process of clotting. During the coagulation time, platelet activation entails enzyme activity and biochemical reactions [32] which may perturb metabolomes otherwise derived from a plasma fraction. Elevated levels of lysophosphatidylethanolamines and lysophosphatidylcholines have been observed in serum [22], which is in line with platelet-induced enzyme activity [22,23,25,28,33]. Reports also suggest that serum metabolite profiles contain a greater absolute analyte count [34], which may be due to the release of additional metabolites from the cell fraction into serum as a result of enzymatic activity or hemolysis.

RT exposure during coagulation is another procedural requirement for serum preparation that may impact metabolite concentrations [16,17]. RT exposure permits persistent cellular metabolism amongst erythrocytes, white blood cells, and platelets, which remain intact during this phase of serum preparation. As such, greater analyte counts [34] and elevated metabolite concentrations [23] may be produced as a result of persistent anaerobic cellular metabolism during serum preparation. Diminished levels of pyruvate [27,28] and glucose [35], as well as elevated lactate concentrations [26,28], have been reported in serum, which is consistent with anerobic metabolic activity during the sample preparation phase. The relationship between blood processing and indicators of anerobic metabolism is discussed in greater detail later in this review.

In contrast to serum, plasma samples retain platelets, resulting in considerations for inter-sample variability. Previous work by Liu et al. [22] showed that the concentrations of 216 metabolites were not discernibly different between standard plasma and cell-free plasma. However, these results are specific to healthy individuals and may not be applicable to patient-derived samples with platelet numbers above the healthy reference limit [22,36]. Given that centrifugal forces within a centrifuge can impact the quantity of platelets present in plasma samples, plasma samples obtained using different centrifugation procedures may not be comparable. Protocols such as that developed by Lacroix et al. [37] may be used to prepare platelet-free plasma (PFP), which may be more suitable for inclusion in metabolomics analyses. PFP preparation requires a two-step centrifugation protocol, with each centrifugation performed at 2500× *g* for 15 min [37].

Studies comparing serum and plasma preparations reported thus far have included relatively small sample sizes. With the exception of two studies, the work summarized in Table A1 reports on samples obtained from fewer than 50 subjects. Additionally, the studies summarized in this section utilize a variety of metabolomics techniques, which may differ in their sensitivity to matrix-dependent metabolome variations. As evidenced in subsequent sections, there is a need for large-scale, standardized analyses to determine the influence of pre-analytical variables on metabolomics measurements. Differences in serum and plasma metabolite quantification should also be evaluated on a larger scale to establish normative concentration ranges for individual metabolites within each biofluid.

### 2.2. Whole Blood and Dried Blood Spot Sampling

Whole blood is a less commonly used in metabolomics research. Unlike serum or plasma, whole blood retains the entirety of the blood cellular compartment, including erythrocytes, white blood cells, and platelets. Previous work has found that the use of whole blood minimizes the risk of hemolysis and allows for a more accurate representation of metabolic pathways compared to serum, including amino acid metabolism, glycolysis, gluconeogenesis, and the urea cycle [38]. Whole blood has been found to contain a greater number of analytes than serum [38], suggesting that it may provide representation of a great proportion of the blood metabolome. Adenosine triphosphate (ATP), succinate, and glutathione are amongst the metabolites identified in whole blood but not in the serum metabolome [38]. Whole blood may be particularly advantageous for analyses aiming to characterize conditions dependent upon red blood cell (RBC) metabolism, such as sepsis [38]. Metabolic markers of dementia [39], aging [40,41], and type II diabetes [42] have previously been identified using whole blood as a platform.

Although whole blood is advantageous from the standpoint of capturing the detailed composition of the metabolome, a lack of metabolism quenching may render the measured metabolome unrepresentative of in vivo blood composition. Following collection, the cellular component of blood remains metabolically active. Under oxidative stress, blood cells utilize anerobic means of energy production and alter the concentration of affected analytes. For this reason, groups should demonstrate caution when selecting whole blood as a matrix for metabolomics work and consider persistent metabolism in the interpretation of their results.

Dried blood sampling is a relatively novel approach to capturing blood metabolome composition. Using this technique, a drop of whole blood is air-dried on filter paper and subsequently extracted using a solvent such as methanol [43,44]. In this paper, we specifically review literature pertaining to fluid blood metabolomics works. As such, dried blood sampling is not considered in the sections that follow.

### 2.3. Recommendations

According to the 2021 International Organization for Standardization (ISO) release, both serum and plasma are acceptable matrices for metabolomics analyses [45]. However, as noted earlier, the differences in metabolite concentration between plasma and serum may vary by up to 20%; therefore, blood and serum are not comparable. Combining plasma- and serum-derived samples for metabolomic testing is likely to introduce substantial variability related to the sample collection process and is not recommended. Therefore, it is important to follow an SOP that specifies acceptable sample types and their appropriate normal reference ranges for metabolomics studies whenever possible. This can facilitate accurate interpretations of the results from various metabolomics studies.

Serum is often considered the “gold standard” for metabolomics work [28,46], as plasma preparation requires the addition of anticoagulative agents, which can interfere with metabolite detection techniques [25,46,47]. Serum preparation is simple and not contingent upon additives and introduces fewer variables for consideration in downstream analysis. As such, many groups favor the use of serum over plasma. From our perspective, the choice of plasma vs. serum samples should be guided by the objective of the analysis. In some circumstances, where prolongation of the potential anaerobic phase is not important, the consistent changes in the fluid-phase metabolome that result from the clotting process may provide important information about a specific pathophysiological process. In such cases, a rigorously collected serum sample may be preferred. However, a plasma sample that has been sufficiently depleted of platelets provides a truer representation of the fluid phase that was in circulation at the time the sample was taken. In instances where the research question does not favour a particular biofluid, the important consideration is to consistently use the same type of sample.

## 3. Pre-Processing Factors Impacting Metabolome Composition

The time following blood collection and prior to centrifugation constitutes the pre-processing phase of blood sample preparation. The choices of blood collection tube, time delay prior to centrifugation, and storage temperature are important factors that must be tightly controlled when preparing samples for metabolomics analyses.

### 3.1. Collection Tube Type

Independent of innate differences in plasma and serum composition, differences in sample collection tube type for both plasma and serum can introduce variability that may become apparent in downstream analysis. Additives present in blood collection tubes have the potential to bias metabolomic profiles by introducing chemical noise into measurements.

#### 3.1.1. Sample Collection Tubes: Serum

The measured composition of the serum metabolome is influenced by the type of collection tube used, with varying metabolite concentrations being observed in serum samples obtained using different coagulants [22]. Thrombin, silicate, and non-additive collection tubes are amongst the most common blood collection tubes used in serum preparation (Table A2). In an analysis conducted by Liu et al. [22], thrombin, silicate, and additive-free tubes were found to produce different concentrations of the dipeptide phenylalanylphenylalanine (Phe-Phe), lysophosphatidylcholine C18:0, and carnitine C2:0. Blood samples were collected from 12 healthy volunteers and processed according to manufacturer instructions. Clotting times for each tube type were as follows: 5 min for thrombin tubes, 30 min for silicate tubes, and 60 min for additive-free tubes—all at RT and followed by centrifugation. Quantitative ultra-high-performance liquid chromatography–mass spectrometry (UHPLC-MS) analysis of samples revealed that Phe-Phe was differentially represented across tubes, with the highest concentration being observed in silicate serum tubes (on average 0.6 μg/mL) and the lowest concentration being observed in thrombin tubes (on average 0.1 μg/mL). The concentration of lysophosphatidylcholine C18:0 was lowest in thrombin tubes and highest in the non-additive tubes maintained at 30 °C for 60 min. Carnitine C2:0 was similarly represented in all serum tubes, also with the highest concentrations in the non-additive tubes maintained at 30 °C for 60 min. In addition to suggesting that the blood collection tube type can influence the detected metabolite concentrations, these findings also identify the sensitivity of blood metabolites to RT exposure and processing time-delays. This is important to consider, as different blood collection tube types require different durations of exposure to RT.

Previous work has also found that serum samples obtained using conventional plastic tubes and serum tubes containing a polymeric gel are not comparable. López-Bacon et al. [48] identified a clear separation between serum and serum–gel samples using principal component analysis (PCA). These authors found that the concentrations of nine metabolites were significantly higher in conventional tubes compared to tubes with polymeric gel, while the concentrations of two metabolites were higher in tubes with polymeric gel (*p* < 0.05). Metabolites related to the citric acid cycle, such as aconitic acid, amino acids (alanine and proline), and aminomalonic acid, were amongst the metabolites shown to differ between conventional and polymeric gel samples. The fold-change difference between conventional and polymeric–gel concentrations ranged from −38X (aconitic acid) to −1.6X (lactic acid). Given these reported differences, it is likely that the inclusion of polymeric substances in blood collection tubes has a significant interfering effect on metabolite measurements.

#### 3.1.2. Recommendations

There are limited data on the impact of coagulants used in different blood collection tubes on serum metabolite measurement. Where there are data available, the biochemical interactions between coagulant additives and metabolomics assays result in differential detection of serum metabolites. Investigators are cautioned against the use of gel tubes for serum preparation due to the possibility of interference by the polymeric gel [48]. Serum is a unique derivative of blood and can be prepared without the addition of coagulant additives. For the purposes of metabolomics research, it is best practice to avoid introducing measurement bias by utilizing non-additive serum collection tubes. When additive-containing tubes are used, the type of collection tube should remain consistent across an analysis to minimize attributable variability in metabolite measurement. Results from analyses where different tube types are used should be compared with caution. The type of blood collection tube used for sample collection should be routinely reported in the research methods for publication.

#### 3.1.3. Collection Tube Type: Plasma

In contrast to serum preparation, blood collection tubes for plasma preparation contain anticoagulant agents, which prevent the onset of blood coagulation by targeting coagulation-inducing proteins and ions. Heparin, citrate, and ethylenediaminetetraacetic acid (EDTA) are most commonly used in clinical practice (Table A3). Heparin-based anticoagulants inhibit thrombin and Factor X, thereby preventing the formation of fibrin, which is required for coagulation [49]. Lithium heparin and sodium heparin plasma tubes are both commonly available and frequently used for clinical blood collection. Citrate-based anticoagulants, such as acid citrate dextrose and sodium citrate, sequester calcium ions required for the coagulation cascade and platelet activity [50,51]. EDTA is functionally comparable to citrate and chelates calcium ions to prevent coagulation and platelet activity.

We identified 16 primary analyses that considered the influence of blood collection tube type on plasma metabolite measurements [17,25,28,34,46,47,48,52,53,54,55,56,57,58,59,60]. Differences in plasma metabolite patterns and the number of detected features can be observed between heparin-, citrate-, and EDTA-based plasma preparations. Specific concentration differences between heparin-, citrate-, and EDTA-based plasma preparations were found to range from subtle to large [17,25,34,46,47]. Amino acids [25], carboxylic acids [25], sugar alcohols [25], acylcarnitines [34], nucleotides [34], trimethylamine-N-oxide [28], and sarcosine [28,55] are amongst the plasma analytes shown to differ according to blood collection tube type. Select metabolite species, including cholesteryl esters ChoE (18:2), ChoE (20:4), diacyl glycerol DG (36:3), and triglyceride analytes may achieve comparable detection across collection tube types. A few discernible trends related to collection tube selection are described below.

According to the existing body of literature, citrate and EDTA plasma types are unsuitable for metabolomics analyses due to measurement biases induced by cationic interactions. Nuclear magnetic resonance (NMR) [28,46,61], ultra-high-performance liquid chromatography–electrospray ionization–quadrupole-time of flight/mass spectrometry (UPLC-ESI-QTOF/MS) [25], LC-MS [53], and ESI-MS [47] measurements are impacted by their use. Citrate and EDTA can alter the ionization efficiency of specific metabolites in MS-based metabolomics work, resulting in misrepresentation of their abundance. Work conducted by Sotelo-Orozco et al. [46], Vignoli et al. [28], and Barton et al. [61] has confirmed that EDTA and citrate collection tubes also produce interfering peaks in NMR analysis.

Sodium citrate is the most commonly utilized citrate-based anticoagulant and has been found to induce ion suppression or enhancement of metabolites co-eluting with citrate peaks [25]. Uric acid, L-tyrosine, uridine, l-pyroglutamic acid, and adenine are amongst the polar metabolites impacted by the use of citrate collection tubes [25]. Additional concerns related to the use of citrate plasma were raised by González-Covarrubias et al. [47], who report that the ionic strength and pH of sodium citrate are not ideal for lipid extraction in LC–ESI-MS analyses and can result in ion suppression of phosphocholines, sphingomyelins, and triglycerides. Glycoprotein and lipoprotein levels have also been reported to be reduced in citrate plasma preparations [28], whilst acylcarnitine concentrations are approximately 20% lower in citrate compared to EDTA plasma [34]. Importantly, the concentration of citrate is higher in citrate plasma compared to EDTA plasma [34,53], with some reports suggesting that citrate concentrations are 34 times higher in samples collected using citrate-based anticoagulants [34] compared to EDTA plasma. This is significant, particularly for analyses aiming to profile citrate, an important biological mediator.

Both dipotassium and tripotassium EDTA salts are commonly available for anticoagulation purposes. Of the two, K3EDTA is not recommended for use due to its effects on blood cell volume [62]. Similar to citrate plasma preparations, EDTA has established effects on NMR and MS-based quantification methods [25,28,46,47,53,61]. Barri et al. [25] noted that K2EDTA separated from citrate and heparin plasma preparations in PCA analysis and that uric acid, L-tyrosine, methionine, and adenine detection are impacted by the use of EDTA in MS readings. Unfilled EDTA collection tubes have been found to contain high levels of sarcosine, making them ill-suited for analyses aiming to capture sarcosine levels [28,55]. O’Rourke et al. noted that elevated levels of hemoglobin were observed in K2EDTA plasma, which is consistent with RBC hemolysis [58]. This finding was absent in matched sodium citrate plasma samples but suggests that the captured metabolite profile in EDTA may be fundamentally altered by hemolytic activity.

Plasma tubes containing heparin as an anticoagulant are available in the form of sodium heparin, lithium heparin, or ammonium heparin tubes [63]. Multiple groups have reported that heparin-based collection tubes are well suited for plasma metabolomics [25,52,64]. Barri et al. [25] confirmed that minimal interference is observed with use of lithium heparin plasma collections in UPLC-ESI-QTOF/MS. Zhou et al. [52] also reported that heparin plasma yielded optimal detection of analyte functional groups, making it an advantageous platform for metabolomics research. It should be noted that heparin plasma use is not without its own complications. In their analysis, Gonzalez-Covarrubias et al. [47] reported that in addition to sodium citrate and potassium EDTA, lithium heparin can also bind to negatively charged phospholipids, enhancing their detection. Additional work has also found that lithium heparin tubes enhance the ionization of phospholipids and triglycerides, modifying metabolite profiles captured by liquid chromatography/tandem mass spectrometric assays [65]. Yin et al. [17] noted that chemical noise was present in the ion chromatograph of lithium heparin, which was later identified as polyethylene glycol clusters. The authors noted that lithium heparin tubes contain plastic beads, which may introduce this measurement interference. Thus, groups seeking to quantify potentially impacted metabolites should still exhibit caution when selecting heparin plasma for use.

#### 3.1.4. Recommendations

According to ISO 23118, EDTA plasma is best suited for both NMR- and MS-based metabolomics work [45]. Heparin-based anticoagulants should be avoided for experiments using NMR to quantify metabolite concentrations [45] but may be acceptable for MS. According to the literature summarized in this section, considerations exist for both EDTA and heparin plasma in metabolite quantification. Neither is evidently the superior type of collection tube for metabolomics analyses. Therefore, both EDTA- and heparin-based plasma collection tubes may be considered for inclusion in metabolomics analyses. The same type of collection tube should be used consistently to minimize attributable variability in plasma metabolite measurement. Results of analyses where different tube types are used should be compared with caution. The type of blood collection tube used for sample collection should be routinely reported in the research methods for publication.

### 3.2. Pre-Centrifugation Sample Handling

The environmental conditions of clinical laboratory testing can affect the stability of blood samples. Delays in separation of the cellular components (leukocytes, erythrocytes, and platelets) from the fluid phase post blood collection permits ongoing cellular metabolism, particularly under RT conditions. In this section, we discuss the impact of processing time delays and temperature conditions during the pre-centrifugation period.

#### 3.2.1. Persistent Cellular Metabolism

Under normal physiologic conditions, energy production to sustain cellular metabolism depends on oxidative phosphorylation (aerobic metabolism). In short, pyruvate produced through glycolysis diffuses into the mitochondria and enters the citric acid cycle, resulting in the production of nicotinamide adenine dinucleotide (NADH) and flavin adenine dinucleotide (FADH2) [66]. These coenzymes enter the electron transport chain, which requires oxygen to act as the final electron acceptor to generate energy in the form of ATP. When oxygen is not available (anaerobic metabolism), pyruvate is instead converted to lactate, which promotes the regeneration of NAD+ from NADH and is permissive of continued glycolysis. Energy production via glycolysis is much less efficient, generating only 2 ATP molecules per molecule of glucose, compared to 32 ATP molecules generated through oxidative phosphorylation [66]. The immediate impacts on the metabolome are the depletion of glucose, pyruvate, acetyl-CoA and the accumulation of lactate. However, given the interconnectedness of these metabolites with multiple other metabolic pathways, the metabolomic perturbations with time are widespread.

RBCs are unique in that they lack mitochondria, making them reliant on anerobic metabolic processes [66]. After blood collection, all of the remaining cellular constituents revert to anaerobic metabolism due to hypoxia. Prolonged exposure of blood samples to RT conditions inevitably leads to the consumption of glucose and the accumulation of lactate, making these metabolites sensitive markers of persistent cellular anaerobic metabolism prior to centrifugation. With depletion of glucose as an energy substrate, cellular stress instigates the consumption of a variety of other metabolites for the purposes of energy production. Cells aim to maintain a high ATP/ADP ratio by inhibiting anabolic functions that consume ATP whilst upregulating alternative pathways to generate ATP [67]. For example, amino acids can become deaminated, and the intermediates used for energy production [68]. Cells may also metabolize lipid molecules to generate energy, particularly when glucose stores are depleted. Fatty acids feed into the citric acid cycle through β-oxidation [67], thereby regenerating glucose stores and ATP. Lipases may convert triacylglycerols into additional fatty acids and glycerol [67]. To obtain an accurate representation of the fluid fraction of the blood metabolome at the time of sampling, it is, therefore, essential to minimize the conditions that promote persistent cellular stress and anaerobic metabolism until the cellular fraction can be separated.

In the next section, we describe trends from primary analyses that considered the influence of pre-centrifugation environmental conditions on blood metabolite concentrations. The main findings are summarized in Table A4. Plasma and serum were found to be similarly susceptible to modification by the pre-analytical environment and are, therefore, discussed together. Evaluated processing time delays ranged from 5 min to 7 days post blood draw, coupled with either storage at RT or cold storage.

#### 3.2.2. Time Delay

Metabolites that are sensitive to delays during processing at RT for four or more hours include lactate [69,70,71,72], glucose [69,70,71,72], pyruvic acid [73], amino acids [17,69,70,74], fatty acids [69], choline [69], acetone [69], zinc [75], hypoxanthine [17,73], lipid-based molecules [17], insulin [76], c-peptide [76], hypoxanthine, sphingosine 1-phosphate (S-1-P), and linolenyl carnitine [17]. Lactate and glucose levels continue to change for the ensuing 6–18 h [69,71]. Among the most significantly altered metabolites were hypoxanthine, sphingosine 1-phosphate (S-1-P), and linolenyl carnitine, which increased by between 7 and 18 fold in a similar time period [17].

Perturbations to the blood metabolome are expected to commence immediately post blood collection and may be most pronounced during the first hour after collection. One study conducted at RT reported an increasing trend in lactate levels within five minutes (0.1 mmol/L increase), with a progressive increase after 30 min (0.36 mmol/L increase) [77]. A larger study (*n* = 63 serial samples) identified a similar trend with, an increase in lactate level of 0.20 mmol/L (14%) after 15 min when subgroups were pooled, although these changes were not statistically significant [78]. It is noteworthy that samples were placed on ice during preprocessing and that the rate of lactate increase diminished greatly after 6–9 min, likely corresponding to the time required for the sample to cool. These findings were not supported by Trezzi et al. [79], who did not identify differences over 10-, 30-, and 60-minute delays using samples from three volunteers. However, Trezzi et al. [79] did report that 48/236 metabolites differed in concentration between samples kept at RT for 1 h and those kept for the same duration on wet ice. This effect persisted for three metabolites after adjustment for multiple comparisons, namely for lactic acid (1.6-fold increase), ascorbic acid (0.10-fold decrease), and unidentified compound RI 1809.71 (0.84-fold change). A difference in the ratio of lactic acid to ascorbic acid was evident after only a 30 min pre-centrifugation delay. The greatest differences in energy substrate concentration are observed in the first hour, with slower rates of change when the preprocessing time is extended beyond two hours [27]. Glucose, ornithine, and cysteine were found to be rapidly depleted after 1 h of incubation, while pyruvate, glycerate, and α-ketoglutarate were elevated [27].

#### 3.2.3. Pre-Centrifugation Temperature

Whereas prolonged processing delays at RT are undesirable, cooling blood samples prior to centrifugation may mitigate the deleterious effects by slowing cellular metabolic processes. Refrigeration, ice water, and cold-pack cooling have been successfully used for this purpose [17,69,71,73,77,79,80,81,82,83]. Compared with RT storage, wet ice cooling exhibits differences, mitigating changes from anaerobic metabolism within as little as 30 min [79]. Using immediate cooling with wet ice (4 °C), several studies have demonstrated that the measured metabolome is not substantially altered for one to six hours before centrifugation [17,69,77,79,80,81]. An analysis conducted by Fliniaux et al. [71] reported that no significant metabolite changes were observed after storage at 4 °C for 24 h compared to a 4 h delay at either RT or 4 °C.

However, it is evident that cooling may not completely mitigate anaerobic effects. Kamlage et al. [80] found that while wet ice cooling for 2 h significantly reduced the number of affected metabolites compared to RT storage; however, 44 metabolites were significantly different relative to samples with no pre-processing delay. Longer durations of cold storage were found to result in a greater number of changes in metabolite concentrations, emphasizing the need to minimize cold storage duration where possible.

Changes in lactate levels are likely the most sensitive measure of persistent metabolism and may be detectable within as little as 30 min [77]. Seymour et al. [77] reported that following a 30 min delay, lactate concentration increased by 0.08 mmol/L (95%CI 0.02–0.13) and 0.18 mmol/L (95%CI 0.07–0.28) when stored in wet ice and on an ice pack, respectively (*n* = 5). The mean difference in lactate elevation between wet ice and ice-pack storage failed to reach statistical significance. However, these findings warrant additional investigation using a larger cohort to determine whether wet ice or ice-pack storage is better suited to prevent disruptions in lactate concentrations. It should also be noted that certain metabolites, including hypoxanthine [73] and zinc [75], have been found to be altered after extended time delays, regardless of storage temperature.

#### 3.2.4. Avoiding Hemolysis in Blood Sample Preparation

Hemolysis occurs when the cell membrane of RBCs is disrupted, resulting in the release of intracellular components into the plasma, including hemoglobin, enzymes, and metabolites [84,85]. Hemolysis may occur in vivo or ex vivo [85] and is frequently encountered in clinical blood sample collection [85,86,87]. Hemolyzed samples may display a pink-to-red tinge [84,88], but a visual cue is not always present [89]. Incorrect needle size, prolonged use of a tourniquet, excessive shaking of samples, delays in processing, exposure to extreme temperatures, and prolonged storage may all produce varying degrees of hemolysis [85,90,91,92,93]. As such, adherence to standard procedures for phlebotomy and sample-handling conditions are essential to maintain RBC integrity.

The intracellular concentrations of blood analytes can be over 10 times higher than extracellular concentrations [84,94], which can substantially affect the metabolite composition of the compartment intended for sampling. Additional, variability may result from differences in the grading and designation/exclusion of hemolyzed blood samples, which may contribute to some of the variation in metabolite concentration trends observed in the literature [89]. Several studies have enumerated the many different metabolites that are impacted by varying degrees of hemolysis [17,80,95]. Those most impacted include acylcarnitine, glycerophospholipid, sphingolipid, sugar, amino acid, and Krebs-cycle intermediate classes of metabolites [95]. Identifying and segregating samples with hemolysis is, therefore, essential at the end of the pre-processing phase to ensure that results are reliable.

#### 3.2.5. Recommendations

According to ISO 23118, pre-centrifugation handling of blood specimens should be limited to a duration of 30 min at RT [45]. Guidelines are not provided for cold storage duration, but the ameliorating effects of pre-centrifugation cold storage are evident in the summarized literature. Where immediate processing is not possible, best practice is to promptly place blood samples into wet ice or subject them to refrigeration upon collection. If samples cannot be immediately cooled, they should be processed or refrigerated within 30 min of sample collection. Serum samples that require RT exposure for clotting should still be maintained on ice or refrigerated after collection and exposed to RT only when clotting and processing can occur successively. Even with cold storage, pre-centrifugation time delays should be minimized to mitigate impacts on some metabolites from anaerobic metabolism. Pre-processing refrigerated storage time should be limited to a maximum of 6 h. If pre-centrifugation delays exceeding 6 h are encountered, samples should be assessed for quality during metabolomic testing, which may include measurement of sensitive metabolites such as glucose, lactate, and ascorbic acid. Pre-processing storage time and cooling procedures should be reported.

Standard operating procedures should be in place to minimize hemolysis during sample collection and processing. Samples should be evaluated for visible signs of hemolysis, and samples with obvious hemolysis may need to be excluded from analysis. There are insufficient data to recommend measurement standards for free hemoglobin, since reference limits for significant hemolysis are lacking.

## 4. Centrifugation Conditions

Centrifugation of whole blood is required to separate serum and plasma supernatant from erythrocyte, leukocyte, platelet, and protein sediment. Centrifugation duration, speed, and temperature parameters must be optimized to balance short turnaround time for preparation and gentle cell handling. Excessive or insufficient centrifugation force or duration results in the contamination of the serum and plasma supernatant due to cellular lysis or the retention of cellular components, respectively. Continued cooling during the centrifugation phase may be required to prevent the progression of enzymatic processes.

Currently, there are no consistent recommendations regarding centrifugation conditions for either plasma or serum metabolomics. Ammerlaan et al. [96] suggest that for serum, the optimal centrifugation protocol is 10 min at 2000× *g*, using medium force of brake. For plasma, the optimal centrifugation protocol was found to be 20 min centrifugation at 2000× *g*, using medium force of brake [96]. Using the validated protocols, >95% of detected metabolites demonstrated an insignificant difference between samples from a given donor. Reproducibility of protein profiles, microparticle counts, and deoxyribonucleic acid (DNA) and hemoglobin concentrations were also incorporated into protocol validation. Notably, a robust recommendation for centrifugation temperature could not be established. Instead, the group emphasizes the importance of utilizing consistent centrifugation temperatures in sample preparation. In their summary article, Kirwan et al. [97] suggested that centrifugation of serum and plasma samples should take place at 2500× *g* or 2750× *g* for 10 min at RT according to findings from Helmholtz Zentrum München and the UK Biobank, respectively.

Centrifugation force impacts the quantity of residual platelets retained in plasma samples. Lesche et al. [98] confirmed that NMR metabolite profiles and platelet concentration differed between EDTA plasma samples centrifuged at 1500× *g* for 10 min or 3000× *g* for 5 min at 20°. This was associated with a higher/lower glutamine concentration, which may be attributable to ongoing platelet metabolism, since glutamine is an oxidative substrate for human platelets [99]. Other studies have not identified significant differences in either serum [69] or plasma [69,100], including work by Liu et al., which included a comparative evaluation of concentrations of 216 metabolites between standard plasma and cell-free plasma [22].

### Recommendations

According to ISO 22138, centrifugation should occur within a minimum of 30 min of blood collection and be conducted in accordance with documented protocols and standardized across analyses [45]. The standard highlights the importance of removing all cellular components in plasma preparation [45], suggesting that platelet-free preparations are most appropriate for plasma metabolomics analyses. Platelet-free plasma can be prepared by conducting two sequential centrifugation steps at 2500× *g* for 15 min at RT.

When developing centrifugation protocols for blood metabolomics work, speed and duration should be determined by manufacturer guidelines for the collection tubes included in the analysis. Speeds exceeding 2750× *g* and durations exceeding 20 min should be avoided to prevent the compromise of sample quality. Centrifugation parameters, including temperature, should be kept constant across analyses to optimize comparability of data and should be routinely reported in the research methods for publication.

## 5. Post-Centrifugation Processing Factors Impacting Metabolome Composition

Sample handling during the post-centrifugation phase may also impact downstream metabolome analysis. Here, the term “post-centrifugation phase” refers to the time period following serum or plasma separation and leading up to metabolomics analysis via MS or NMR. Samples intended for biobanking are aliquoted and frozen at −80 °C after centrifugation. The near-complete absence of cellular components at this stage means there is little further concern related to anaerobic metabolism. However, improper handling, including time delays, RT exposure prior to freezing, and repeated freeze–thaw cycles, may continue to affect metabolite stability.

### 5.1. Sample Stability over Time

Long delays post centrifugation and before freezing may be consequential, especially when they occur at RT. Previous analyses have assessed delays ranging from 0.5 to 24 h post blood separation [69,80,101]. Bernini et al. [101] examined a range of storage times and identified differences in serum and plasma NMR profiles within 6 h of RT exposure following processing, with greater concentration changes observed in serum samples. Triglycerides, proline, choline, citrate, histidine, albumin, and LDL/VLDL were amongst the metabolites (and proteins) demonstrating sensitivity to post-centrifugation delays. Lipids and sphingolipids (glycerol, lysophosphatidylcholine (C17:0), etc.), carbohydrates (maltose, glycerate, etc.), and amino acids (glutamate, leucine, taurine, proline, etc.) are affected after longer exposure times, exceeding 16 h [80]. Shorter delays of 15 min to 1 h at RT have little impact and may be acceptable [69] for both serum and plasma, although Kamlage et al. [80] identified slight differences in four plasma metabolites after only 30 min at RT.

### 5.2. Temperature

Time delays and storage temperatures likely have a combined effect on metabolite measurements. As with the pre-centrifugation phase, sample cooling has a protective effect on sample stability [80,102,103]. Anton et al. [102] reported that serum metabolite concentrations were significantly altered when maintained at RT for 12 h compared to samples cooled on wet or dry ice. In their analysis, they used targeted flow injection analysis–electrospray ionization–triple quadrupole mass spectrometry (FIA-ESI-MS/MS) to measure 127 metabolites in serum samples exposed to handling delays of 12, 24, and 36 h at RT on wet and dry ice. The group found that changes in 18 metabolites were significantly associated with both the time and temperature coefficients in a mixed-effects linear regression model (*p* < 1.3 × 10^−4^), but no metabolites were associated with time alone. This suggests that time delays post centrifugation are only impactful with the added effect of RT storage. A total of 22 metabolites showed concentration differences within 12 h of RT storage delay, including analytes in the acylcarnitine, lysophosphatidylcholine, phosphatidylcholine, and amino acid classes. Storage on wet ice resulted in metabolite changes after 24 h of storage, but concentration changes were less pronounced compared to RT storage. Barton et al. [103] also found that the serum metabolome remained unperturbed following a post-centrifugation delay of 24 h at 4 °C. Delays exceeding 24 h result in detectable differences in metabolome composition, even if coupled with cold storage [103].

Notably, even during shorter post-centrifugation delays, cold storage minimizes metabolome perturbations but does not prevent changes altogether. Kamlage et al. [80] reported that following a two-hour delay, 7 plasma metabolites were found to be altered when maintained at 4 °C, while 28 metabolites showed a change in concentration when maintained at RT for the same duration. As such, post-centrifugation time delays, either with cold storage or without, should be minimized or avoided altogether for complete preservation of the original blood metabolome.

### 5.3. Recommendations

ISO 23118 does not provide guidelines for time delay or storage temperature conditions following centrifugation of blood samples [45]. However, the organization does emphasize the need to document conditions during processing and leading up to freezing. Considering the summarized results in tandem, it is recommended to freeze serum and plasma samples immediately post centrifugation. Cold storage provides an ameliorating effect but should be limited to durations less than 2 h for maximum sample quality and accurate representation of metabolome composition. If longer delays are encountered, a validation study on the fridge stability of blood samples is recommended for the metabolites of interests. Delays at RT should be avoided for blood samples intended for use in metabolomics analysis.

### 5.4. Freeze–Thaw Cycles

The number of freeze–thaw cycles a sample undergoes may influence analyte stability and the overall composition of the measured metabolome. This may be largely due to processing and storage parameters during the thawing process until the residual sample is refrozen.

Three different studies have identifies that four to five freeze–thaw cycles are not associated with any significant change in metabolite concentrations [17,71,102], with changes thereafter being more noticeable in metabolites with low molecular weight [71]. In two analyses, samples were thawed at RT for either 30 or 60 min, and in one analysis samples were allowed to thaw in ice water for an unknown duration of time. All samples were refrozen at −80 °C between cycles. Two other studies have identified perceptible differences with three or more freeze–thaw cycles [104,105]. Both studies were conducted using samples that were thawed at RT for either 90 min or an unknown duration of time and refrozen at either −70 or −80 °C. Variation in time and temperature of procedures to both thaw and then refreeze samples may explain some of the differences in these reports. Given the impact of time and RT on metabolite stability in the post-centrifugation phase, it is reasonable to infer that similar constraints should be applied.

### 5.5. Recommendations

According to the ISO 23118 guidelines for metabolomics research, blood samples must be thawed on ice prior to measurement [45]. Best practice is to begin experimental procedures immediately following sample thawing. The unused portion of the sample should be kept refrigerated after it has been aliquoted and until it is refrozen, should be done as soon as possible. The number of freeze–thaw cycles should be limited to two unless there are additional quality assurance procedures in place to confirm that the metabolite measurements are reliable, with further freeze–thaw cycles occurring thereafter.

## 6. Sample Quality Control Markers

Evaluation of sample quality is an important initial step in analyzing metabolomic data from blood samples. There are different approaches reported, and they typically are directed toward identifying the extent of changes resulting from anaerobic metabolism. In this section, we review work that has identified potential markers of pre-analytical variability related to sample-handling conditions.

Elevated lactate and diminished glucose levels are the primary indicators of persistent anerobic metabolism. Jobard et al. [69] assessed the ratio of lactate:glucose as a marker of extended RT handling during the pre-centrifugation phase. Marked differences were observed in lactate and glucose concentrations in serum and plasma samples kept at RT for 6 h prior to centrifugation. The group compared the lactate:glucose ratio and orthogonal partial least square discriminant analysis (OPLS-DA) models based on global NMR metabolic signatures as markers of sample quality. The OPLS-DA metabolite model demonstrated a clear distinction between samples maintained at RT for 3 h compared to samples maintained at RT for 6 h in serum and plasma. Receiver operating characteristic (ROC) curve analysis showed that the area under the curve (AUC) for the OPLS-DA signature was 0.99 and 1.0 in serum and plasma, respectively, while the AUC values for the lactate:glucose ratio were 0.91 and 0.94, respectively. The model equation and diagnostic threshold for the lactate:glucose concentration ratio is required to separate samples according to quality but was not provided by the study authors. Nonetheless, this study highlights the performance of the lactate:glucose ratio as a marker of sample quality that warrants further exploration. More recently, Jain et al. [106] reported on the use of multivariate patterns to identify metabolite profiles indicative of sample quality. The group showed that a condensed dataset of only 68 metabolites could be combined in a PCA model to accurately identify samples left at RT for over 20 h. They also found that samples experiencing pre-centrifugation time delays ≤ 1 h or ≥4 h could be identified using a two-step approach, with identification criteria comprising (1) an ornithine/arginine ratio ≥1.41 × median and (2) three out of four markers (5-oxoproline, lactate, pyruvate, or fumarate) ≥1.41 × median. For each individual predictor outlined in this approach, a threshold of 1.41 × the median was found to limit the number of false positives and accurately identify samples delayed for 4 or more hours.

Trezzi et al. [79] developed the “LacaScore”, which is the concentration ratio of lactic acid to ascorbic acid multiplied by 10^5^, as a marker of improper pre-centrifugation handling and sample quality degradation. Their results showed that a pre-centrifugation delay of 1 h at RT significantly altered the NMR spectra of the plasma metabolome and that lactic acid and ascorbic acid were the two most altered metabolites, increasing and decreasing at RT compared to on ice. Subsequent testing showed that the “LacaScore” decreased over time between 30 min and 3 h at both 4 °C and RT. The authors suggested that decreasing ascorbic acid levels may be due to degradation or conversion to oxalate [79,107]. Thus, by combining increasing lactic acid levels with decreasing ascorbic acid levels, the “LacaScore” captures both the enzymatic activity accompanying cellular metabolism and the degradation of metabolites due to pre-analytical environmental conditions. This may be preferred when studying disease states where abnormal glucose or lactate concentrations reflect an underlying pathology as opposed to blood sample quality. The final LacaScore algorithm outlines the following thresholds: <5.2 corresponds to <3 h at RT or ≤3 h at 4 °C during the pre-centrifugation phase, 5.2 ≤ LacaScore ≤ 52 is an indeterminate quality score, and >52 corresponds to ≥3 h RT or >3 h 4 °C pre-centrifugation delay. Independent application of this model to two additional plasma cohorts yielded diagnostic accuracies of 71% and 86% [79].

Anton et al. observed that amino acids and lipids could serve as markers of metabolomic sample quality [102], given the significant changes in concentration observed with post-centrifugation RT handling. Their analysis revealed that samples maintained at RT for a minimum of 12 h post centrifugation underwent distinct metabolic changes; amino acids and lysophosphatidylcholines were increased, while phosphatidylcholines were decreased. A total of 235 metabolite ratios were found to be associated with time delays and RT exposure. Random forest analysis and classification tree models were identified that could accurately predict the RT exposure of a serum sample based on the ratio of total lysophosphatidylcholine concentration to total phosphatidylcholine concentration and the ratio of glutamine concentration to serine concentration. Notably, these models predict post-centrifugation handling conditions, unlike the previously described lactate:glucose [69] and “LacaScore” [79] models. It is also important to highlight that acylcarnitine, amino acid, glycerophospholipid, and sphingolipid analytes were overrepresented in the assay panel, limiting the identification of sample quality markers to these metabolites. Hexose concentrations were quantified but not found to be associated with sample quality.

## 7. Recommendations for Bio-Banked Sample Labelling

To encourage complete documentation of biospecimen handling during the pre-analytical phase, the International Society for Biological and Environmental Repositories Biospecimen Science Working Group published a global standard code for bio banked samples [108]. The Sample PREanalytical Code (SPREC) outlines 7 important pre-analytical variables to be included in the label of fluid biospecimens and 13 variables for solid biospecimens. Using this abbreviated coding system, sample type, primary container, pre-centrifugation time delay and temperature, centrifugation speed and temperature, second centrifugation speed and temperature, post-centrifugation delay, and long-term storage vessel and temperature are delineated using a three-letter code. For example, a double spun, sodium-EDTA plasma sample stored at 3 to 7 °C for <2 h pre-centrifugation centrifuged at RT for 10 min twice, maintained at 3 to 7 °C for <1 h post centrifugation, and stored at −85 to −65 °C for long-term storage may be labelled as PL2-SED-B-B-E-A-G. Such labelling procedures should be encouraged at biobank facilities to standardize pre-analytical handling procedures and facilitate detailed reporting of sample handling in analytical reports. Broad adoption of standardized pre-analytical handling procedures will also ensure reproducibility and the inter-comparability of metabolomic analyses.

## 8. Summary

Biobanks adhere to strict collection and handling procedures for samples they curate for future research. Given the emergence of metabolomics as one of the important ‘omics’ to understand human health and disease, it is important to ensure that sample handling and processing parameters used in biobanking processes are optimized to ensure that such samples can be reliably used for metabolomic testing.

Sample type, pre-centrifugation time and temperature, post-centrifugation time and temperature, and the number of freeze–thaw cycles prior to analysis must be controlled to avoid post-collection modification of blood metabolite concentrations. For some metabolites, the impact of deviation from optimal parameters may be minor, but for others, the change in concentration can be substantial. This is most important when investigators are seeking to identify more subtle perturbations and may be less relevant when log-fold changes in some metabolites are being evaluated. Nonetheless, establishing such standards that can be broadly applied can help to ensure that metabolomic test results are readily comparable between programs and that the most valuable information can be extracted from these samples.

We have referenced here existing standards and provided additional recommendations based on available literature. We also reviewed some practical approaches to evaluating sample quality in the analysis phase after testing. Many of the studies referenced herein were small in size and, therefore, somewhat limited in their power to characterize small differences related to sample processing. Additional studies are needed to address some of the areas where data are incomplete to make definitive recommendations. Pre-analytical factors, including sample collection tube type, maximum permissible pre- and post-centrifugation time delays, and centrifugation conditions, must be evaluated further to develop standardized protocols for blood metabolomics analyses.

## Data Availability

Not applicable.

## Appendix A

**Table A1:** Differences in metabolome composition between serum and plasma.

Author	Year	Methods	Main Findings
Liu et al. [22]	2018	K+-EDTA plasma vs. non-additive serum UHPLC-MS *n* = 10 (20 plasma and serum samples)	Acyl-carnitines, FFA, fatty acid amides, LPC, LPE, PC, PCO, PE, and SM differed between plasma and serum No significant differences for most amino acids, bile acids, medium- and long-chain acyl-carnitines, and several phospholipids
Yu et al. [23]	2011	*n* = 377 (754 plasma and serum samples) Targeted analysis 163 metabolites	104 metabolites showed higher concentrations in serum Arginine, diacyl-phosphatidylcholine C38:1, LPC (C16:0, C17:0, C18:0, and C18:1), serine, phenylalanine, and glycine > 20% greater in serum
Denery et al. [24]	2011	*n* = 12 (24 plasma and serum samples) Heparin plasma vs. serum LC-ESI-MS	Charged peptides and peptide fragments higher in serum Lysophosphatidylinositol higher in plasma
Kaluarachchi et al. [26]	2018	*n* = 8 HILIC and RP UPLC-MS	Samples clustered according to donor, not sample type The methyl group of lipids, the methyl group of lactate, and the methylene group of glutamine were higher in serum Glucose higher in plasma than serum 47 lipoprotein subclasses differed between serum and plasma samples
Wedge et al. [30]	2011	*n* = 29 patients (58 plasma and serum samples) GC/TOF-MS analysis and UHPLC/MS Analysis	Sulfate, dipropylacetic acid, leucine, serine, methionine, arachidonic acid, alpha-glucopyranosiduronic acid, citric acid, cellobiose, and eicosanoic acid were only present in serum 2-methylsuccinic acid, α-hydroxybutyric acid, aspartic acid, 4-hydroxyproline, 3-phenylpropanoic acid, tyrosine, α-linolenic acid, urea, and citrulline were only present in plasma α-Hydroxybutyric acid and tyrosine showed more variation in plasma Tryptophan, galactose, malonic acid, leucine, and serine showed more variation in serum
Kennedy et al. [34]	2021	*n* = 27 Whole blood, serum, lithium heparin, potassium EDTA, and sodium citrate plasma LC/MS/MS analyses	Serum contained the most measurable analytes Strong separation between the whole-blood and serum/plasma samples Lithium heparin and serum similar
Liu et al. [27]	2010	*n* = 15 (30 plasma and serum samples) GC/TOF MS analysis	More variance in plasma samples 29 metabolites were lower in plasma, including most amino acids, carbohydrates, hypoxanthine, glycerol-3-phosphate, and β-hydroxybutyrate Pyruvate, citrate, glycerate, fumarate, and nitrogen metabolites urate and hydroxylamine were higher in plasma
Barri et al. [25]	2013	*n* = 21 (3 plasma samples, 1 serum sample per patient) UPLC-ESI-QTOF/MS analysis	Serum contained the most analytes Peptides differed between serum and plasma samples
Vignoli et al. [28]	2022	*n* = 6 (6 × 3 = 18 samples) Citrate plasma, EDTA plasma, and serum tubes NMR	Higher concentrations of alanine, glutamine, glycine, histidine, leucine, N,N-dimethylglycine, phenylalanine, tyrosine, and valine in serum High lactate and low pyruvate in serum Acetone, acetic acid, and formic acid lower in serum Different metabolite inter-relationships in serum and plasma
Bovo et al. [31]	2023	*n* = 24 pigs Targeted analysis Biocrates AbsoluteIDQTM p180 kit; 186 metabolites	High mean correlation coefficient between plasma and serum samples 33/44 metabolites higher in serum Amino acids and acylcarnitines higher in serum Arginine, C10:2, putrescine, taurine, and serotonin >20% higher in serum
Suarez-Diez et al. [29]	2017	*n* = 1000 Either plasma or serum provided NMR Additional study; *n* = 377 FIA–MS/MS	Different metabolite associations between serum and plasma PCA showed separation of serum and plasma, even when derived from the same sample 23/29 metabolites higher in serum Ormate, pyruvate, alanine, threonine, and oxoglutarate higher in plasma

FFA—free fatty acid, LPC—lysophosphatidylcholine, LPE—lysophosphatidylethanolamine, PC—phosphatidylcholine, PCO—ether phospholipid, PE—phosphatidylethanolamine, SM—sphingomyelin, LC-ESI-MS—liquid chromatographic−electrospray ionization mass spectrometric analysis, HILIC—hydrophilic interaction chromatography, RP UPLC-MS—reverse-phase ultra-high-performance chromatography, GC/TOF-MS—gas chromatography–time-of-flight mass spectrometry, UHPLC/MS—ultra-high-performance liquid chromatography–mass spectrometry, NMR—nuclear magnetic resonance, FIA–MS/MS—flow injection analysis–tandem mass spectrometry.

**Table A2:** Characteristics of common serum collection tubes.

Blood Matrix	Tube Additive	Clotting Time	Impact on Metabolomics Testing
Serum	Thrombin	5 min	Lowest concentration of Phe-Phe [22] Lowest concentration of lysophosphatidylcholine C18:0 [22]
Serum	Silicate	30 min	Highest concentration of Phe-Phe [22]
Serum	Non-additive	60 min	Highest concentration of lysophosphatidylcholine C18:0 [22] Highest concentration of carnitine C2:0 [22]

**Table A3:** Characteristics of plasma collection tubes.

Anticoagulant	Anticoagulation Mechanism	Considerations for Metabolomics Work
Citrate	Calcium chelator	Ion suppression and enhancement [25,47] Interfering NMR peaks [28,46,61] Elevated levels of citrate [34,53]
Ethylenediaminetetraacetic acid (EDTA)	Calcium chelator	Ion suppression and enhancement [25,47] Interfering NMR peaks [28,46,61]
Heparin	Thrombin inhibition	Enhanced ionization of phospholipids and triglycerides [47,65] Noise in mass spectrometry readings [17]

**Table A4:** The impact of pre-centrifugation time and temperature on blood metabolite measurements.

Author	Year	Time Conditions	Temperature Conditions	Matrix	Significant Findings	Recommendations for Storage
Jobard et al. [69]	2016	1 or 6 h time delay	RT or 4 °C (fridge)	Plasma and serum	↑ Lactate, ↓ glucose with increased delay and temperature Fatty acids, choline, acetone, and alanine (serum only) changed	<6 h at RT; 6 h at 4 °C acceptable
Liu et al. [27]	2010	1–4 h	37°	Plasma and serum	↓ Glucose, ↓ amino acids, ↑ pyruvate, ↑ lactate, and ↑ TCA intermediates	<1 h at 37°; most changes occur during first 2 h of delay
Nishummi et al. [73]	2018	RT: 0, 15, or 30 min Cold storage: 1, 4, or 8 h	RT or cold storage (Cube Cooler, Forte Grow Medical, Tochigi, Japan)	Plasma and serum	Sucrose levels did not change with cold storage, but pyruvic acid levels decreased with longer cold storage Hypoxanthine levels increased with pre-centrifugation delays, regardless of storage temperature	<30 min at RT; 1 h cold storage acceptable
Nkuna et al. [76]	2023	8, 12, 48, and 72 h	RT	Plasma and serum	C-peptide and insulin levels changed within 8 h	<8 h at RT
Ghini et al. [70]	2022	30 min–72 h Protocols from multiple biobanks considered	RT or cold storage	Plasma and serum	↑ Lactate and ↓ glucose associated with time delays between 30 min and 4 h at RT, as well as 72 h at 4 °C	<30 min time delay at RT; <72 h at 4 °C
Wang et al. [82]	2018	0, 15, 30, and 48 h	Cold storage (refrigerator)	Plasma	Majority of metabolites were stable for up to 48 h Nucleotides, energy-related metabolites, peptides, and carbohydrates showed changes within a 15 h delay	<15 h cold storage
Breier et al. [74]	2014	3 h, 6 h, and 24 h	RT and cold storage (cool pack)	Plasma and serum	Majority of analytes were stable for 24 h at RT and under cold storage for both plasma and serum Changes in amino acids and biogenic amines detected after 3 h of cold storage	<3 h cold storage
Fliniaux et al. [71]	2011	4 h or 24 h	RT or 4 °C	Serum	↑ Lactate and ↓ glucose with increased delay and temperature 24 h pre-centrifugation delay did not change the metabolite profile compared with 4 h delays at RT or 4 °C	<4 h at RT; 24 h at 4 °C is acceptable
Yin et al. [17]	2013	2, 4, 8, and 24 h	RT and cold storage (ice water)	Plasma	64 analytes changed with increased RT exposure No significant changes in any individual sample for up to 4 h of cold storage Hypoxanthine, sphingosine 1-phosphate, and linolenyl carnitine most affected	<2 h at RT; up to 4 h cold storage is acceptable
Trezzi et al. [79]	2016	10, 30, 60 min, 3 h, or 23 h	RT or 4 °C	Plasma	Storage temperature has a larger impact than time delay alone 48/236 metabolites affected by storage temperature and 3/236 by time delay ↑ Lactate and ↓ ascorbic acid in RT vs. cold-storage samples	<3 h cold storage
Xiong et al. [93]	2024	24 h	RT or 4°C	Plasma	Haemolysis detected in samples stored at RT but not at 4 °C Sodium fluoride tubes prevented changes in glucose and lactate levels for 24 h at 4 °C and RT	Up to 24 h at 4 °C
Killilea et al. [75]	2024	1 h, 4 h, or 24 h	4 °C, 20 °C, or 37 °C for 1 h	Plasma and serum	Zinc concentration changes observed after 4 h, regardless of storage temperature	<4 h at RT or cold storage
Liu et al. [22]	2018	2 h or 4 h	RT or 4 °C (ice water)	Plasma	RT storage resulted in large concentration changes, while cold storage resulted in fewer changes ↑ Lactate in the samples kept at 4 °C for 4 h	<4 h cold storage
Seymour et al. [77]	2011	0, 5, 10, 20, and 30 min	RT, cold storage 1 (ice pack), or cold storage 2 (wet ice)	Plasma	↑ Lactate in RT samples compared to samples kept on wet ice or ice pack within 5 and 10 min, respectively	<5 min at RT; cold storage on wet ice preferred
Jones et al. [78]	2007	0, 3, 6, 9, 12, and 15 min	RT or cold storage (on ice)	Plasma	Lactate concentrations did not significantly change over 15 min either at RT or under cold storage	15 min at RT or cold storage
Clark et al. [83]	2004	1, 2, 3, 4, and 7 days	RT or 4 °C	Plasma	Alpha-carotene, beta-carotene, lutein, lycopene, retinol, and alpha-tocopherol changed by <8%, and cryptoxanthin and gamma-tocopherol changed by <11% at RT and under cold storage	RT and/or cold storage for several days is permissible
Kamlage et al. [80]	2014	2 h or 6 h	RT or cold storage (wet ice)	Plasma	Cold storage resulted in fewer metabolite concentration changes 44 metabolite concentrations changed after storage on wet ice for 2 h	<2 h cold storage
Sens et al. [81]	2023	20, 60, 120, and 240 min	RT or cold storage (ice water)	Plasma	Metabolite concentration changes most drastic in RT samples Ethanol amines, acylcarnitines, nicotinamide, and lactic acid amongst the most-affected metabolites	1 h cold storage
Debik et al. [72]	2022	30 min or 1, 2, 4, or 8 h	RT	Plasma and serum	Lipoproteins minimally affected by RT exposure Changes in lactic acid, glucose, glutamic acid, and more seen within 1 h of RT storage	<1 h RT storage

**Table A5:** Summary of recommendations.

Analytical Phase	Recommendation
Choice of matrix	Serum or plasma acceptable Consistent sample type used throughout analysis
Serum collection tube	Non-additive tube
Plasma collection tube	Heparin or EDTA anticoagulants Consistent collection tube used throughout analysis
Pre-centrifugation time and temperature	RT exposure limited to 30 min Up to 6 h delay permissible with cold storage or refrigeration Pre-centrifugation conditions reported
Centrifugation	Centrifuge within 30 min of blood collection Avoid speeds >2750 g and durations >20 min Centrifugation protocols should be consistent across analyses
Post-centrifugation time and temperature	Limit RT exposure post centrifugation Up to 2 h delay post centrifugation permissible, with cold storage Freeze samples (−80 °C) as soon as possible following processing
Freeze–thaw cycles	Limit freeze–thaw cycles to 2 Thaw on ice
